# Universality of the *Phytophthora* mating hormones and diversity of their production profile

**DOI:** 10.1038/s41598-017-05380-3

**Published:** 2017-07-10

**Authors:** Tomohiko Tomura, Shylaja D. Molli, Ryo Murata, Makoto Ojika

**Affiliations:** Graduate School of Bioagricultural Sciences, Nagoya University, Nagoya, 464-8601 Japan

## Abstract

Sexual reproduction of the plant pest *Phytophthora* is regulated by two mating hormones α1 and α2, which are acyclic oxygenated diterpenes first isolated from *P. nicotianae* A1 and A2 mating types, respectively. A previous report suggested the universality of these factors within this genus. To confirm this concept, we investigated 80 strains (19 species) of *Phytophthora* and a related genus, not only for the responsiveness to mating hormones but also for their productivity. The results indicated that among the 55 heterothallic strains, 24 (44%) responded to a mating hormone and 40 (73%) produced one or both hormones. These findings demonstrate the interspecies universality of mating hormones within the genus *Phytophthora*. Hormone productivity was found to be highly diverse and dependent on the strains used. Although the A2 mating type has been regarded as the α2 producer, 19 (59%) of the 32 A2-type strains produced both the hormones and two A2-type strains exclusively produced α1 in high yields. These results indicate that hormone biosynthesis in *Phytophthora* is universal but highly diverse and complex, and varies with culture conditions, providing us valuable information for future studies on the mechanism of mating hormone biosynthesis of *Phytophthora*.

## Introduction

**Figure 1:**
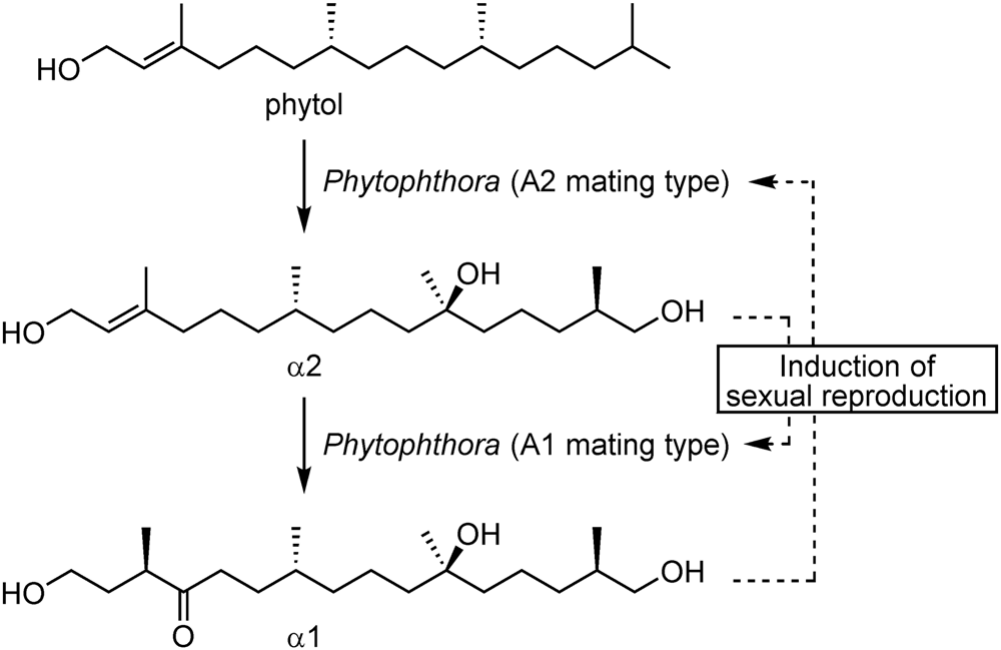
Mating hormones and induction of sexual reproduction in the plant pathogen *Phytophthora*. Regarding the coexistence of two mating types of *Phytophthora*, the A2 mating type converts phytol to α2, which is then converted to α1 by the A1 mating type. The secreted mating hormones α2 (from A2) and α1 (from A1) induce the sexual reproduction of the counter mating types A1 and A2, respectively.

The genus *Phytophthora* represents a group of filamentous fungus-like oomycetes and includes more than 100 species, most of which are deleterious to a broad range of economically and ecologically important plant species^1, 2^. In this genus, *P. infestans* is notorious as the causal agent for the Great Famine during the mid-1840s, which wiped out the entire potato crop in Ireland and eventually led to mass starvation^2^. Sexual reproduction is one of the most important biological events occurring during the lifecycle of species in this genus, where the female and male organs fuse and are fertilized to produce sexual spore oospores. The heterothallic (self-sterile) members require pairing of two compatibility types, A1 and A2, whereas the homothallic members can produce oospores even in a single culture^3^. Evidence of hormonal regulation during *Phytophthora*’s sexual reproduction was reported by Ko, and these hypothetical factors were termed α1 (secreted by A1) and α2 (secreted by A2)^4^. It is believed that each mating type responds to the hormone secreted by the counter mating type to form oospores, providing advantages such as a sustainable structural nature (double-walled sporangia) and the potential of accelerated evolution^5^. Therefore, research on the mechanisms behind *Phytophthora*’s sexual reproduction is key to controlling this agricultural pest. After many years of efforts, the mating hormones α1 and α2 were identified from an A1 mating-type strain of *P. nicotianae* in 2005^6^ and from a strain of the counter A2 mating type in 2011^7^, respectively. Both hormones, characterized as new acyclic diterpenes, were found to be biosynthesized from phytol by incorporation experiments (Fig. 1)^7^. It was reported half a century ago that most interspecies pairing between heterothallic species of A1 and A2 types resulted in sexual reproduction^3^. In addition, the isolated hormones induced sexual reproduction of certain other species^6, 7^, suggesting the interspecies universality of these mating hormones.

The aim of the present study is to validate the concept that hormones are the universal mating factors within this genus, by demonstrating both responsiveness to hormones and their productivity. This was achieved using 80 strains (19 species) of *Phytophthora* and the related genus *Halophytophthora* (comprising halophilic members of *Phytophthora* that predominantly inhabit brackish water mangroves^8^).

## Results

### Mating type analysis

Some species of *Phytophthora* are homothallic and capable of conducting sexual reproduction (producing oospores) in single cultures. However, other species are heterothallic and require the pairing of different mating types for conducting sexual reproduction^9^. There are three mating types (or “induction types” as proposed by Ko^10^) known in the heterothallic species of this genus, depending on the productivity of, and responsiveness to, the mating hormones: (1) A1, (2) A2, and (3) A1,A2. The A1 mating-type strains are those that produce α1 (to induce oospores in the counter mating type A2) and/or respond to α2 produced by A2), whereas the A2 strains are those that produce α2 (to induce oospores in the counter mating type A1) and/or respond to α1 (produced by A1). The A1,A2-type strains were defined by Ko^10^ as those that either produce both the mating hormones or respond to both hormones, although it is a hypothetical type without examples. The A0-type strains neither stimulate others nor are stimulated by any mating-type strains to produce oospores.

**Figure 2:**
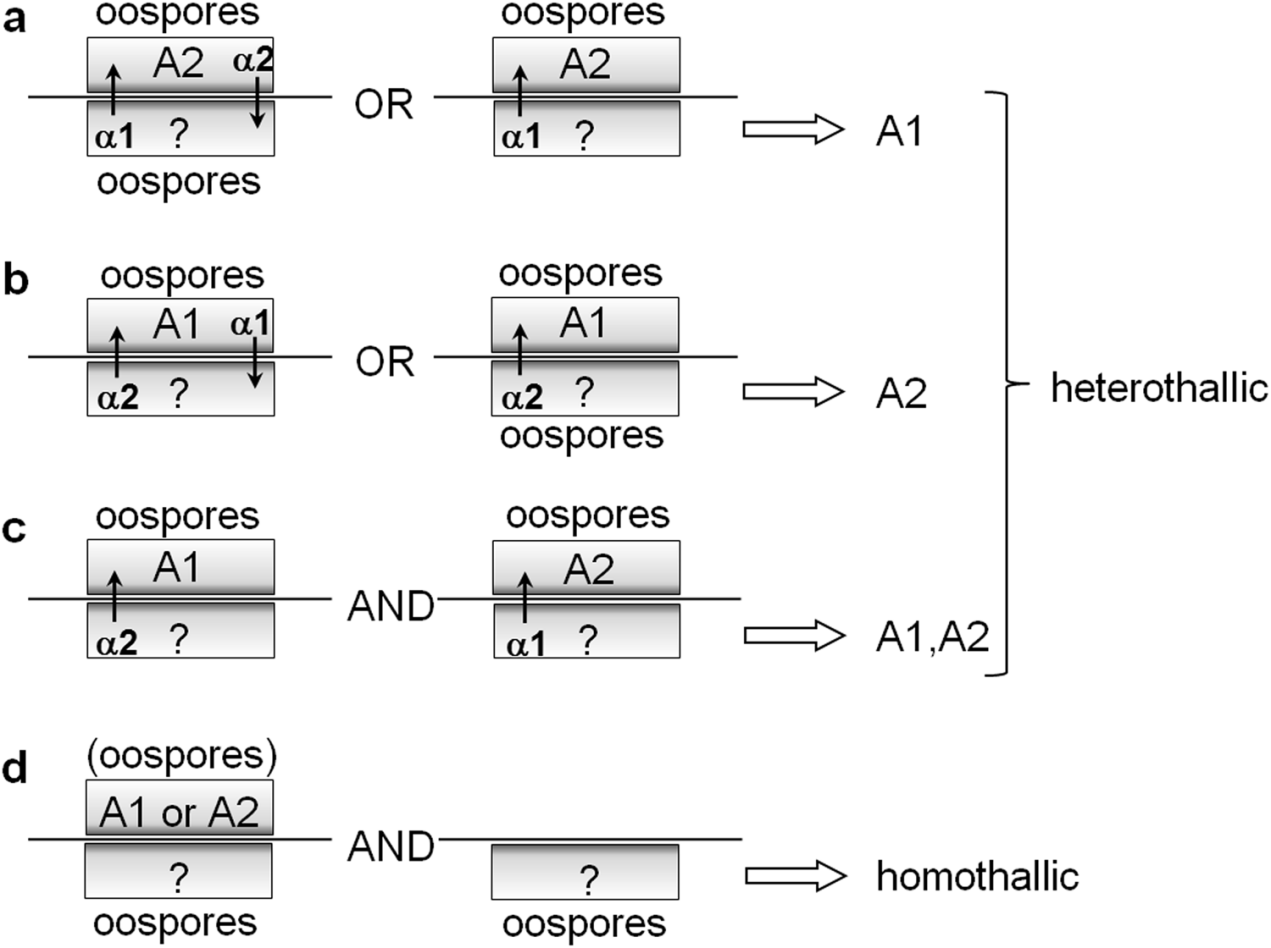
Evaluation of the mating types of *Phytophthora*. The evaluation is based on the hormone production/reception experiment reported previously^11^. A tested strain (down) was paired with the tester strain (up) *P. nicotianae* American Type Culture Collection (ATCC) 38607 (A1 mating type) or ATCC 38606 (A2) on the opposite side of a polycarbonate membrane (horizontal bars) to avoid direct hyphal contact. (**a**) The strains that induced the oospores of the A2 tester were assigned as A1, which may or may not produce oospores in response to the A2 tester. (**b**) The strains that induced the oospores of the A1 tester were assigned as A2, which may or may not produce oospores in response to the A1 tester. (**c**) The strains that formed no oospores but induced oospores in both the A1 and A2 testers were designated as A1,A2. (**d**) The strains that produced oospores in the presence and absence of a tester strain were assigned as homothallic species, which may or may not induce oospores in the tester strains.

The mating types of our *Phytophthora* library (80 strains), consisting of 71 strains of *Phytophthora* and 9 strains of *Halophytophthora*, were evaluated by a polycarbonate membrane test (Fig. 2, supplementary Table S1) as described by Ko^11^. The results enabled us to newly assign the mating types of 46 among the 76 strains purchased from the Biological Resource Center, National Institute of Technology and Evaluation (National Biological Research Center (NBRC), Chiba, Japan). In addition, the evaluation resulted in the revision of the mating types of nine strains (Table 1) and the discovery of seven strains of the A1,A2 type, which is a hypothetical induction type reported previously^10^.

**Table 1:** Response to and production of mating hormones in the genera *Phytophthora* and *Halophytophthora*.

Species	Strain^c)^	Mating type^d)^	Response to^g)^		Production of (μg L^−1^)^h)^	
			α1	α2	α1	α2
*P. cactorum*	NBRC30474	Homo^e)^			ND	0.6
	32191	Homo^e)^			ND	0.4
	32192	Homo^e)^			ND	ND
	32193	Homo^e)^			ND	ND
	32194	Homo^e)^			ND	ND
*P. cambivora*	30471	A1	−	−	0.9	ND
	30472	A2	+	−	0.5	0.2
*P. capsici*	8386	A2^e)^	−	−	26.7	24.2
	30696	A1	−	+	1.4	ND
	30697	A2	+	−	3.3	0.5
	30698	A2	+	−	34.8	0.4
	30699	A1^f)^	−	−	ND	ND
	31400	A1,A2^f)^	−	−	0.4	ND
	31402	A1	−	+	ND	ND
*P. cinnamomi*	33180	A1	−	+	10.2	ND
	33181	A1,A2^f)^	+	−	29.0	0.6
	33182	A1	−	+	14.6	ND
	33183	A1,A2^f)^	−	−	69.3	5.3
*P. citrophthora*	31408	A0^f)^	−	−	ND	ND
	31410	A1,A2^f)^	−	−	ND	ND
*P. colocasiae*	30695	A2	+	−	6.7	0.6
*P. cryptogea*	31411	A1^e)^	−	−	ND	ND
	31412	A1^e)^	−	−	1.2	ND
	31622	A2^e)^	−	−	21.2	ND
	32325	A1,A2^f)^	−	−	10.7	ND
	32326	A2	−	−	65.3	ND
*P. humicola*	32771	Homo^e)^			ND	ND
*P. infestans*	9173	A1^e)^	−	−	ND	ND
	9174	A1,A2^e)^	−	−	0.5	0.3
	PI0-1	A1^e)^	−	+	0.3	ND
	PI1234-1	A2^e)^	+	−	3.5	19.2
*P. katsurae*	9753	A2^e)^	−	−	ND	ND
	30433	A2^e)^	−	−	ND	ND
	30434	Homo^e)^			ND	ND
	30435	Homo^e)^			ND	ND
*P. megasperma*	31624	Homo^e)^			ND	ND
	32174^b)^	Homo			ND	ND
	32175	Homo			ND	ND
	32176	Homo			ND	ND
*P. melonis*	31413	A1^f)^	−	+	1.1	ND
	31414	A1,A2^f)^	−	−	0.4	ND
	31415	A2	−	−	1.4	5.2
*P*. *nicotianae*	ATCC38606	A2	+	−	2.1	16.8
	ATCC38607	A1	−	+	1.1	ND
	4873^a)^	A2^e)^	−	−	ND	ND
	9049	A2^e)^	+	−	0.8	2.3
	30595^a)^	A2^e)^	+		1.8	10.9
	31416^a)^	A2	+	−	1.0	3.6
	31419^a)^	A2	+	−	0.3	6.2
	31423	A1	−	+	2.1	ND
	31425	A1	−	+	6.2	ND
	33190	A2	+	−	ND	23.6
	33191	A1^f)^	−	+	0.8	ND
	33192	A2	−	−	0.4	19.4
	33193	A2	+	−	0.6	13.2
*P. palmivora*	9755	A2^e)^	+	−	0.7	8.2
	30285	A1^e)^	−	−	0.7	ND
	31428	A1	−	−	ND	ND
*P. porri*	30417	A0^e)^	−	−	NT	NT
	32963	A0^e)^	−	−	NT	NT
*P. sojae*	31014	Homo^e)^			ND	ND
	31015	Homo^e)^			ND	ND
	101543	Homo^e)^			ND	ND
	101544	Homo^e)^			ND	ND
	101545	Homo^e)^			ND	ND
	101546	Homo^e)^			ND	ND
	101547	Homo^e)^			ND	ND
	105919	Homo^e)^			ND	ND
*P. vignae*	30473	Homo^e)^			ND	ND
	30613	Homo^e)^			ND	ND
*Phytophthora sp*.	32716	A1^e)^	−	−	0.8	ND
*Halophytophthora operculata*	32865	A2^e)^	−	−	ND	ND
*Hp. vesicula*	32216	A2^e)^	−	−	ND	ND
	32444	A2^e)^	−	−	ND	ND
	32445	A2^e)^	−	−	ND	ND
	33128	A2^e)^	−	−	ND	ND
	33164	A2^e)^	−	−	ND	ND
	33165	A2^e)^	−	−	0.4	0.2
	33166	A2^e)^	−	−	0.8	0.2
	33265	A2^e)^	−	−	ND	1.5

^a)^Variants (var. *nicotianae*, var. *parasitica*), which are regarded as synonyms in the NBRC catalog, are omitted (see supplementary Table S1 for details). ^b)^Species to be revised on the basis of a recent ribosomal RNA gene analysis by NBRC. ^c)^Numerals indicate NBRC numbers (for instance, 30474 represents NBRC 30474). ^d)^Assigned by a co-culture method with the standard strains *P. nicotianae* ATCC 38606 (A2) and 38607 (A1) (Fig. 2, Table S1). Homo and A0 indicate homothallic (self-fertile) and neuter strains, respectively. ^e,f)^These mating types are newly assigned (e) or revised (f) by the present study. ^g)^The “+” sign indicates a positive response (oospore formation) to a hormone, whereas the “−” indicates no response at the dose of 300 ng per disk. The hormonal response was not tested for the homothallic and A0 strains, which produce oospores without mating hormones and show no response, respectively. ^h)^The sign ND (not detected) indicates an amount below the detection limit (0.13–0.31 μg L^−1^) in LC/MS analysis. The sign NT (not tested) was used for A0 strains.

### Responsiveness to mating hormones

To demonstrate the interspecies universality of the mating hormones, we first evaluated the responsiveness of all 80 strains (19 species) to the mating hormones (Table 1). An α1-adsorbed paper disk or an α2-adsorbed membrane filter was placed on a pre-cultured colony of each strain. Two dosages of hormones, 100 or 300 ng per disk, were used for all strains. After an appropriate incubation time, oospores induced around the disk were observed. The original hormone producers *P. nicotianae* ATCC 38606 (A2 mating type) and ATCC 38607 (A1 mating type) responded to α1 and α2, respectively, at a minimal dose of 3 or 10 ng per disk^6, 7^.

Among the 55 heterothallic strains (13 species) tested, 24 strains (44%) or eight species (62%) responded to one of the mating hormones (Table 2). Since the 22 homothallic strains were self-fertile, even in the absence of hormones, their responsiveness was not examined. These results demonstrate that the mating hormones isolated from *P. nicotianae* are not species-specific, but may contribute to interspecies crossing in the genus *Phytophthora*. Although 31 (56%) heterothallic strains showed non-responsiveness to a hormone, some (17 strains, 31%) can produce the hormone(s), as mentioned in the next section. Although the remaining 14 heterothallic strains (25%) exceptionally showed neither response to nor production of the hormones, this might be due to the difference in the culture conditions. Namely, the mating type was determined under a specific co-culture condition (polycarbonate sandwich method, Fig. 2), whereas the hormone response assay used a disk diffusion method on the solid culture and the hormone production assay was performed by the liquid culture (next section). The discrepancy between the mating type evaluation test and other two chemical tests (hormone response and production assays) will be discussed later.

**Table 2:** Summary of interspecies universality of the mating hormones in *Phytophthora* and *Halophytophthora*.

Mating type	Heterothallic				Homothallic	Neuter (A0)
	A1	A2	A1,A2	Total		
Number of strains (species) tested	18 (8)	30 (11)	7 (4)	55 (13)	22 (6)	3 (2)
Response to: α1	0	13	1	24 (8)	—	0
α2	10	0	0		—	0
Production of: α1	13	2	3	40 (10)	0	—
α2	0	2	0		2	—
both	0	17	3		0	—

The number of species is indicated in parentheses. The number of species in the “total” column does not necessarily agree with the sum of each number of species within the same raw because some species contain different mating type strains. The production of the hormones at the bottom is discussed in the next section in detail.

### Production of mating hormones

**Figure 3:**
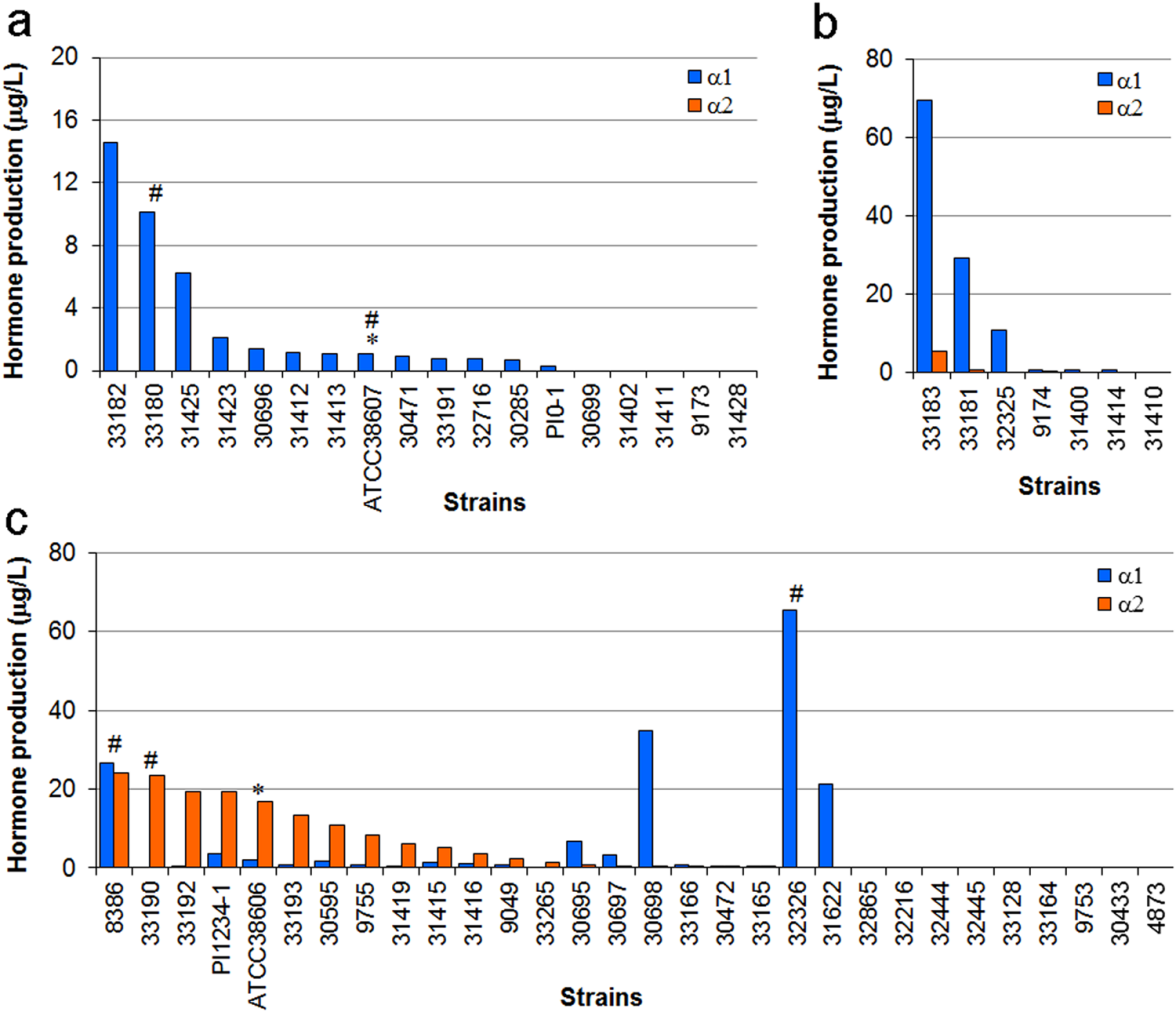
Difference in mating hormone productivity between mating types. The mating hormone production by the A1 (**a**), A1,A2 (**b**) and A2 (**c**) mating-type strains is shown in descending amounts of α1 (**a**,**b**) and α2 (**c**). The numerals without alphabetic characters indicate the NBRC strain numbers. The asterisks (*) indicate the standard strains previously used for the first hormone isolation^6^ and as the mating type testers. The hash signs (#) indicate the strains used to demonstrate the time-dependent production of the hormones (see the next section). Each datum was obtained by a single experiment.

We further confirmed this universality by a quantitative analysis of the hormones produced in the above-mentioned *Phytophthora* and *Halophytophthora* strains using the liquid chromatography/mass spectrometry (LC/MS) technique. Both heterothallic and homothallic strains were cultured for 1–2 weeks in a 20% V8 juice liquid medium supplemented with a piece of A2 mycelia (as the α2 producer for culturing the A1 mating type) or phytol (for culturing the A2 and A1,A2 mating types) to stimulate hormone production^6, 7^. Each culture supernatant was extracted with ethyl acetate, and a portion of the extract was injected into an LC/MS apparatus. Electrospray ionization-time of flight mass spectra (ESI-TOF MS) of α1 and α2 showed characteristic molecular ions and fragmentation patterns. Under the optimized conditions, the high-intensity pseudo-molecular ions of *m/z* 309.3 [M–2H_2_O+H]^+^ for α1 and *m/z* 351.3 [M + Na]^+^ for α2 were used to generate ion chromatographs, and then to obtain standard curves (Fig. S1). Finally, both hormones were detected at 50 pg/injection or higher. Since the portion injected into LC/MS corresponded to 0.64 or 0.8 mL of the culture broth, the detection limits for α1 and α2 were 0.13–0.16 μg L^−1^ (or 0.25–0.31 μg L^−1^ for some exceptional culture conditions), respectively. The amount of hormones secreted into the culture medium is summarized in Table 1 and Fig. 3, showing that 40 (73%) of the 55 heterothallic strains produced one or both hormones (Table 2). The hormone α1 was detected in the liquid cultures of 13 A1 strains (72% of A1), and α2 was not detected in A1 strains (Table 2). In contrast to the A1 strains, 17 A2 strains (57% of A2) produced not only α2, but also α1, and two A2 strains exclusively produced α1. This was a surprise because the A2 mating type was regarded as the α2 producer.

Sexual reproduction of the homothallic species of *Phytophthora* may also be controlled by the mating hormones because most of the homothallic strains induced sexual reproduction of the tester strains (*P. nicotianae* ATCC 38606 and 38607) in the mating type evaluation test (polycarbonate sandwich test, Fig. 2, Table S1). Similar results were previously observed by Ko^11^. However, at least in the liquid medium of V8 juice, the majority of homothallic strains did not secrete the mating hormones; only two of the 22 strains produced α2 in a low yield (Table 1). This contradiction may be due to the difference of the sensitivity of hormone detection and culture conditions between the biological mating type evaluation test and the chemical hormone production experiments as discussed later. The mechanism of sexual reproduction of the homothallic species is an additional attractive issue to be resolved in the future.

### Factors affecting the production of the mating hormone

Although the present study revealed the universality of the *Phytophthora* mating hormones, it also raised several questions regarding hormone production: (1) the A2 mating type is not merely an α2 producer, but a dual hormone producer, which is not illustrated by the biosynthetic route proposed by us (Figs 1 and 2) hormone productivity depends highly on strains, and certain strains have no hormone productivity (Fig. 3), which does not perfectly support hormone universality. These unexpected results may be due to the difference in the optimal culture conditions for each of the strains. Since such information is valuable for studying the mechanism of hormone biosynthesis in the future, we next examined the factors that affect the production of mating hormones.

**Figure 4:**
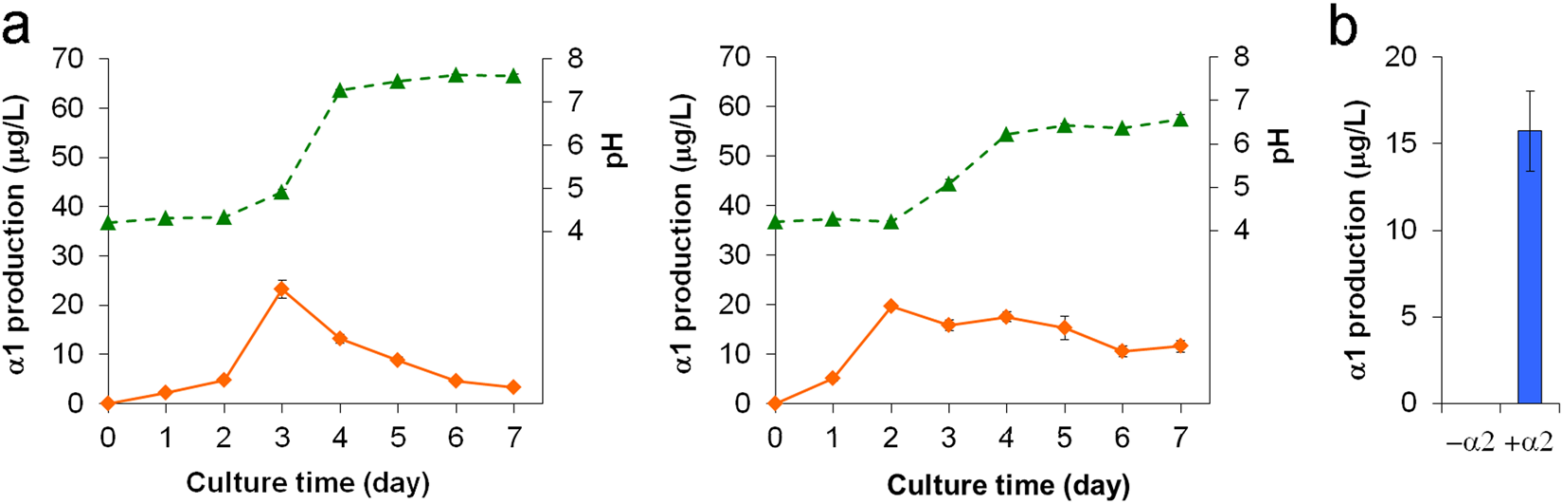
Production by the *Phytophthora* A1 mating-type strains. (**a**) Time-dependent α1 production of two strains (*P. nicotianae* ATCC 38607 and *P. cinnamomi* NBRC 33180) in the V8 juice liquid medium. Solid lines show the α1 production based on LC/MS analysis, and dotted lines show the pH value of the medium. α2 (50 μg L^−1^) added as a precursor of α1. (**b**) *P. capsici* NBRC 31402 showed no production of α1 in the V8 juice medium supplemented with A2 mating-type mycelia, whereas it could produce α1 in the α2 (50 μg L^−1^)-containing 20% V8 juice medium.

To determine the optimal culture time for hormone production, we first analyzed the time-dependent hormone production of the two strains (two species) of the A1 type in the standard liquid medium (20% V8 juice) supplemented with α2 (α1 precursor) through a week by using a shaking incubator. A portion of the culture supernatants was tapped daily, and the mating hormones were quantified by LC/MS (Fig. S2). Two A1 mating-type strains, *P. nicotianae* ATCC 38607 and *P. cinnamomi* NBRC 33180, converted α2 into α1 (Fig. 4a). The production of α1 increased and reached a maximum two or three days after the start of the culture, following which production decreased with a rapid increase in pH (Fig. 4a), which synchronized with the mycelial growth by appearance. Although five A1 mating-type strains did not produce α1 (Fig. 3a), they might have the potential to produce α1 because they induced sexual reproduction in the A2 type tester strain in the mating type evaluation test. Actually, *P. capsici* NBRC 31402 produced α1 (15.7 mg L^−1^) when the 20% V8 juice medium was supplemented with α2 (Fig. 4b). The lowered productivity might be due to the insufficient supply of the precursor α2 in the usual culture conditions used in Fig. 3a. On the other hand, the other 13 strains (Fig. 3a) could produce α1 by using the limited amount of α2 that was supplied by a piece of the A2 strain (α2 producer) added to the 20% V8 juice medium.

**Figure 5:**
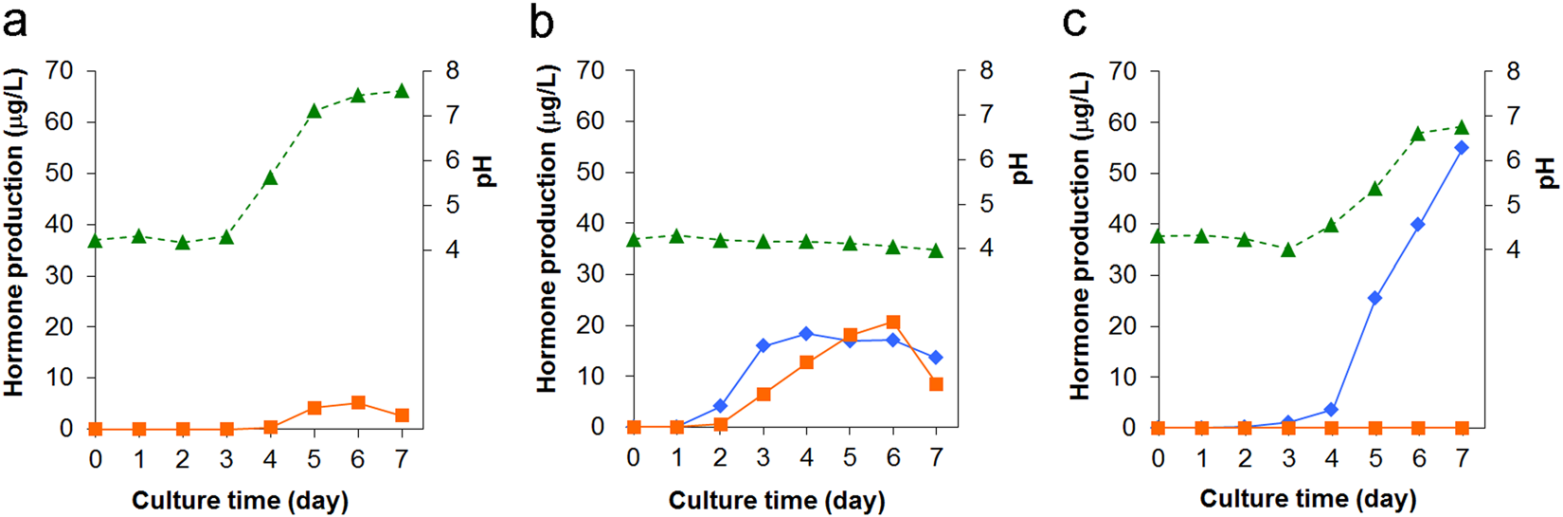
Time-dependent mating hormone production of *Phytophthora* A2 mating-type strains in V8 juice liquid medium. (**a**) A conventional A2 mating-type strain (*P. nicotianae* NBRC 33190) that produced only α2 from phytol. (**b** and **c**) Exceptional A2 mating-type strains that produced both hormones (**b)**, *P. capsici* NBRC 8386), or only α1 (**c**, *P. cryptogea* NBRC 32326). Solid lines with diamond and square labels show the α1 and α2 production, respectively, based on LC/MS analysis. Dotted lines show the pH value.

Hormone production of the A2 mating-type strains in the V8 juice medium is more complex (Fig. 3c) than that of A1 strains, and can be classified into three production patterns: (1) exclusive α2 producers (e.g., *P. nicotianae* NBRC 33190), (2) dual producers (e.g., *P. capsici* NBRC 8386), and (3) exclusive α1 producers (e.g., *P. cryptogea* NBRC 32326). We examined the effect of culture time and media on hormone production. *P. nicotianae* NRBC 33190 produced only α2, which was regarded as the typical nature of the A2 type. However, this type has not been found to be specific in the present study. The α2 production started on day 4 or 5 in synchrony with the rapid pH increase (and with the mycelial growth by appearance) (Fig. 5a). On the other hand, *P. capsici* NRBC 8386 produced not only α2 but also α1, and their production started on day 2 and reached their maxima on days 6 and 4, respectively, without any change in pH (Fig. 5b). *P. cryptogea* NRBC 32326 is fairly unusual because it secreted only α1, despite its A2 nature, as indicated in the mating-type decision test (Fig. 2, Table S1). The α1 production was much higher than that of the A1 mating type, and synchronized with the increase in pH (and mycelia growth) (Fig. 5c). These results indicate that the production pattern of the mating hormones is fairly diverse in every *Phytophthora* species.

**Figure 6:**
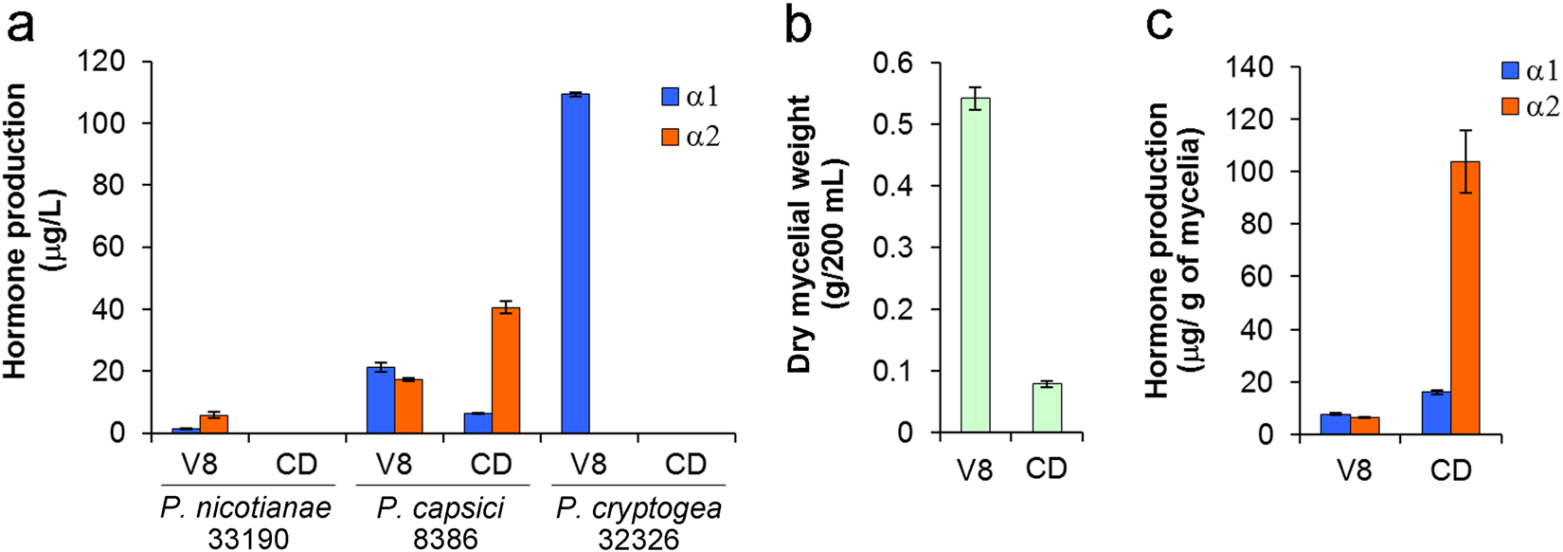
Comparison of the hormone production of three A2 strains in 20% V8 juice and Czapek-Dox (CD) liquid media. (**a**) Mating hormone production of the three indicated A2 strains in μg L^−1^, (**b**) dry mycelial weight of *Phytophthora capsici* NBRC 8386 in gram per flask (broth volume: 200 mL), (**c**) mating hormone production per dry mycelial weight of *P. capsici* NBRC 8386 in μg g^−1^. The strains were cultured for 7 days. Phytol (1 mg L^−1^) was added as the precursor of the mating hormones.

We next investigated the effect of culture media on the production of mating hormones using the three aforementioned A2 strains. These strains were cultured for a week in 20% V8 juice or Czapek-Dox (a general synthetic medium for fungi) liquid medium supplemented with phytol. *P. nicotianae* NBRC 33190 and *P. cryptogea* NBRC 32326 produced the mating hormones in 20% V8 juice, but not in the Czapek-Dox medium (Fig. 6a), confirming that V8 juice is a suitable medium for the production of mating hormones, whereas Czapek-Dox medium is not suitable, at least for these two strains. On the other hand, *P. capsici* NBRC 8386, which comparably produced both α1 and α2 in 20% V8 juice, produced the hormones even in the Czapek-Dox medium (Fig. 6a). Note that the α1:α2 ratio largely shifted to α2 in the Czapek-Dox medium. Since gross hormone production was approximately constant in the two media, V8 juice may contain a promoting factor for α1 biosynthesis from α2. However, we noticed a large difference in the hyphal growth of this strain between the two media: V8 juice medium showed much higher growth promotion than Czapek-Dox (Fig. 6b). Taking this into account, we found that the “α1 production per mycelial weight” (cellular titer of α1 biosynthesis) in the Czapek-Dox medium was comparable to that in V8 juice; however, the “α2 production per mycelial weight” (cellular titer of α2 biosynthesis) in the Czapek-Dox medium was 16-fold that in V8 juice (Fig. 6c). At the cellular basis, the Czapek-Dox medium appears to be considerably superior to the V8 juice medium for promoting the biosynthesis of α2 by the dual hormone producer *P. capsici* NBRC 8386.

**Figure 7:**
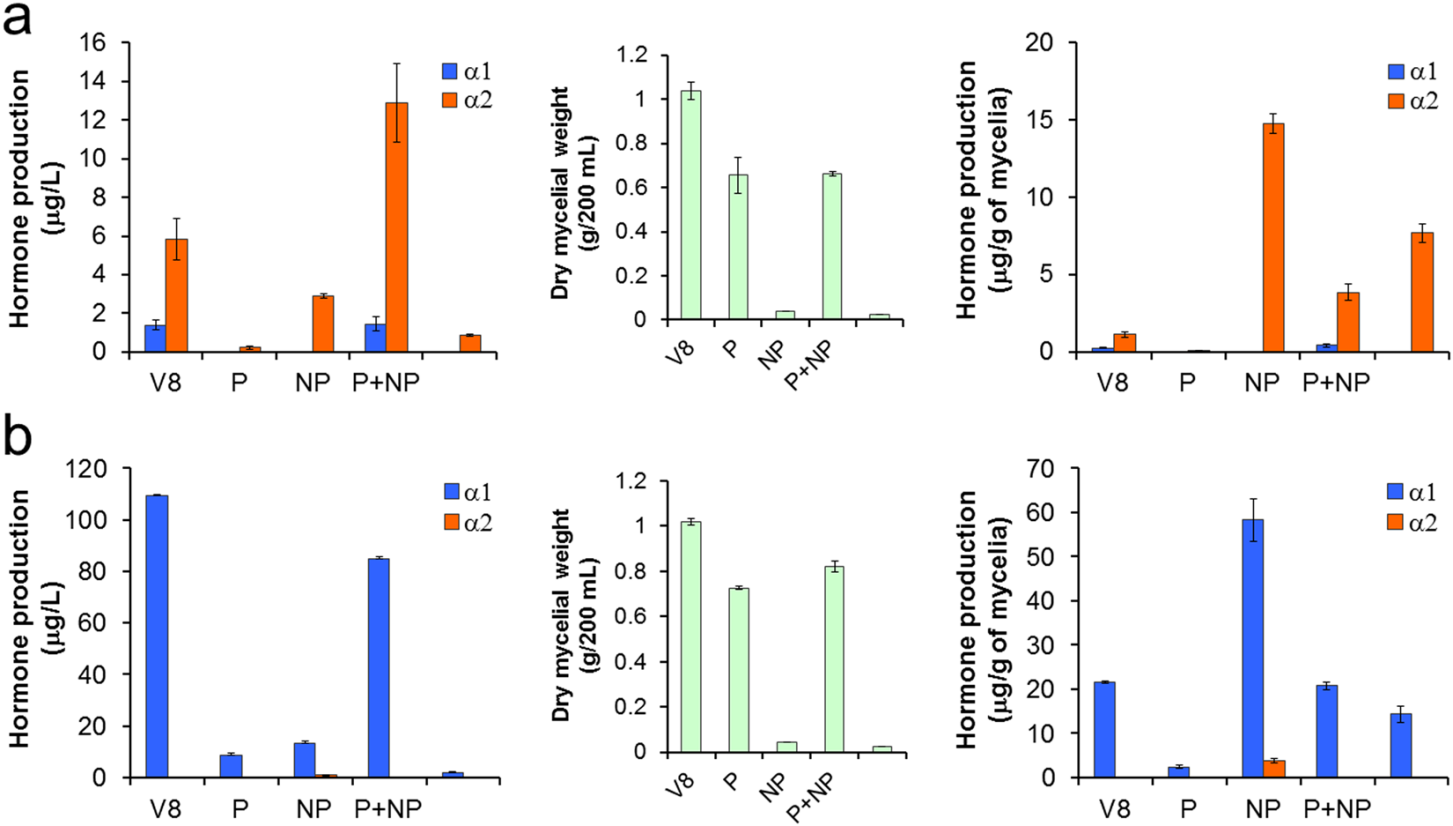
Effect of V8 juice fractions on the mating hormone production. Hormone production of *Phytophthora nicotianae* NBRC 33190 (**a**) and *P. cryptogea* NBRC 32326 (**b**). The left, middle, and right graphs show hormone production (μg) per broth volume (L), dry mycelial weight (g) per flask (broth volume: 200 mL), and hormone production (μg) per dry mycelial weight (g), respectively. V8 indicates 20% V8 vegetable juice medium. P and NP indicate the non-adsorbed (polar) and adsorbed (non-polar) fractions, respectively, in water at a concentration equivalent to 20% V8 juice. No mark indicates the production in only water as a control.

Unlike the hormone production of the dual hormone producer *P. capsici* NBRC 8386, that of *P. nicotianae* NRBC 33190 (a typical α2-producing A2 strain) and *P. cryptogea* NRBC 32326 (an unusual α1-producing A2 strain) was highly promoted in the V8 juice medium compared to the Czapek-Dox (low nutritional) medium (Fig. 6a). Therefore, we next determined which components of V8 juice affected the hormone production of these strains. V8 juice was separated by reversed phase column chromatography into two fractions, namely non-adsorbed (polar, P) and adsorbed (non-polar, NP) fractions, and their solutions in water were used as culture media for these strains. The non-polar fraction (NP) of V8 juice more strongly promoted the α2 production of *P. nicotianae* NBRC 33190 than the polar fraction (P) did (Fig. 7a, left), whereas the fraction P significantly promoted its growth (Fig. 7a, middle), suggesting that non-polar low-nutrient components of V8 juice contain promoter(s) for the α2 biosynthesis. This tendency was more obvious when the hormone productivity per unit mycelial weight was calculated (Fig. 7a, right). The low α2 production in the P medium was restored by adding the lipophilic fraction NP (P + NP medium, Fig. 7a, left), although the reason for the higher total production in P + NP than that in V8 juice is unclear. The α1 production of the exclusive α1 producer *P. cryptogea* NBRC 32326 showed a similar pattern (Fig. 7b). The considerably high hormone production “per mycelial weight” in the NP medium (Fig. 7, right graphs) was due to the low growth of the strains by the removal of nutrients into the water fraction (P) (Fig. 7, middle graphs). A similar effect of nutritional deficiency was observed when the strains were cultured in pure water (Fig. 7, the data without mark). These strains were able to produce the hormones in a moderate yield in terms of the productivity in the cellular base (Fig. 7, right graphs), even though they hardly grew in pure water. These results suggest that there are lipophilic factors in V8 juice that promote the hormone biosynthesis in *Phytophthora* without mycelial growth.

## Discussion

Mating hormones (α1 and α2) are the essential factors that trigger the sexual reproduction of heterothallic members of the plant pathogen *Phytophthora*. Although they were originally discovered from specific strains of *P. nicotianae*, their universality within this genus has remained unclear. In the present study, we examined interspecies universality of the mating hormones by using 80 strains (19 species) of *Phytophthora*, including a related genus (*Halophytophthora*). First, the mating types of all strains were defined by a face-to-face co-culture method (Fig. 2, Table S1), which made it possible to know both the hormone production and hormone response ability of each strain in the same time and contributed to newly assigning or revising the mating types of 55 strains. The mating type revision may be due to the transsexual nature of *Phytophthora*, which was previously reported^12^ and complicated the understanding of the *Phytophthora* sexuality. In this test, we found a wide range of hormone response/production ability of the 55 heterothallic strains as judged from the number of oospores (ranging from a few to thousands, data not shown) formed in the tested (unknown)/tester (standard) strains, suggesting that the responsiveness/productivity of the mating hormones strongly depends on the individual variability of *Phytophthora* strains. Although we set the border at 10 oospores induced in the tested/tester strains to distinguish heterothallic (A1/A2) and neuter (A0) types for convenience, the evaluation heavily depends on how the border is set. Therefore, the mating type of the strains with low hormone response/production ability may be expedient in this study.

Next, we evaluated their hormone responsiveness by a disk diffusion assay with pure mating hormones. The rate of 44% of the heterothallic strains (62% in terms of species, Table 2) was found to be hormone-sensitive strains, which may be insufficient to declare the “interspecies universality” of the mating hormones. This may be caused by the difference of the sensitivity between the mating type definition test (face-to-face co-culture, Fig. 2) and the hormone response test (disk diffusion assay with pure hormones). The former condition could imitate the natural crossing of two mating types, whereas the latter chemical test is unnatural and its sensitivity is much lower than the former test with our experience. For example, the paper disk method in the α2 test resulted in the insufficient oospore formation in A1 type strains, which was the reason why a specific membrane filter was used in the case of the α2 assay. This suggests that the hormone sensitivity could be influenced by the method of hormone administration and there is still room for the improvement of the disk diffusion test to demonstrate the perfect universality in terms of the responsiveness to the purified hormones. In addition, the mating type definition depends on how the border between the heterothallic and neuter strains was set in the test (Fig. 2) as mentioned above. If the border was at a higher level (e.g., 50 oospores), the definition of “heterothallic” could become more distinct, leading to the increase of the rate of the hormone-sensitive heterothallic strains.

On the other hand, the chemical analysis of hormone productivity was performed by using liquid culture conditions. This revealed that 73% of the heterothallic strains (77% in terms of species, Table 2) produced the mating hormone(s), demonstrating the presence of interspecies cross-talk of the mating hormones in *Phytophthora*. The principal reasons why all the heterothallic strains did not produce mating hormone(s) could be the difference of the culture conditions (solid/liquid) and hormone detection methods (biological/chemical) between the mating type definition test and the hormone production experiments. In the mating type definition test (Fig. 2), the hormones produced by tested strains are immediately recognized by the facing tester strain (hormone “receptors”), whereas, in the hormone production analysis, the produced hormones are exposed to a liquid medium for one week prior to the LC/MS analysis. Therefore, the former method should be more sensitive and accurate than the latter one, suggesting that the strains lacking hormone production (27% of heterothallic strains) possibly produce hormone(s) at a level lower than the LC/MS detection limit (as indicated as ND in Table 1). This idea is supported by the fact that *P. capsici* NBRC 31402(A1) lacking the α1 productivity in the standard condition (Fig. 3a) turned out to be a typical α1 producer in the medium supplemented with the biosynthetic precursor α2 (Fig. 4b). This comprehensive analyses revealed the diverse and complicated hormone production profile: (1) the productivity in the standard culture condition (a liquid V8 juice medium, 7 days) greatly depended on the strains used (Figs 3 and 2) unlike the A1 mating type that is the exclusive α1 producer (Fig. 3a), the A2 mating type is not a simple α2 producer but has the potential of the α1 production (Fig. 3c), suggesting that most of the A2 type possess a dual hormone biosynthetic system. In addition to the sensitivity of the hormone detection methods, the former finding (1) could be another reason why some heterothallic strains did not produce enough hormones. Namely, the culture time for the maximum hormone production is not always 7 days but rather varies between 2 and 7 days or longer (Figs 4a and 5), while the comprehensive hormone productivity data (Table 1, Fig. 3) were commonly collected on day 7. These data would be valuable to know an ideal culture time for the maximum expression of the hormone biosynthetic enzymes in the future. The second finding (2) about the dual sexuality of A2 mating type is a strange and interesting in the present research. Three kinds of hormone production patterns (α2, α1, and both) were observed for the A2 mating type, and not only the hormone productivity but also the α2/α1 ratio were affected by the culture media (Fig. 6). Since the A2 mating type strains that produced α1 in the liquid media (especially, *P. cryptogea* NBRC 32326 in Figs 6 and 7) did not start sexual reproduction (oospore formation) alone on the agar media, these strains do not seem to produce α1 in the solid-state. Why can the A2 mating type produce α1 at least in the liquid-state? A putative hypothesis is as follows. The ancient *Phytophthora* all produced both hormones and were self-fertile (or homothallic), and then some strains abandoned (or furnished a suppressor of) the α2 biosynthetic function leading to the creation of the A1 mating type that produce only α1. Further evolution may split the two hormone biosynthetic functions. The fact that the liquid environment is preferred by the motile *Phytophthora* gamete “zoospores” should be considered as another factor to explain the transsexual-like phenomenon because the nature of parents is not always inherited to their progeny in *Phytophthora* ^12^. Since the standard medium (V8 juice) was found to strongly promote the hormone production of some strains compared to a low-nutrient medium (Fig. 6), a preliminary investigation on the promoting factors in V8 juice was performed. The result indicated that the factors could be concentrated into the lipophilic fraction of V8 juice and the growth-promoting factors (nutrients) were removed into the water-soluble fraction (Fig. 7). This would make it possible to obtain *Phytophthora* mycelia that possess a high titer of hormone biosynthesis for the future studies on the hormone biosynthetic mechanism. In summary, the present findings suggest that the individual *Phytophthora* strain demands a specific culture condition for maximum hormone production, and will be valuable for future studies on the mechanism of mating hormone biosynthesis and for a better understanding of the hormonal regulation of *Phytophthora* sexual reproduction, which may provide ideas for novel approaches to control this agricultural pest.

## Methods

### Phytophthora strains

The A1 (ATCC 38607) and A2 mating-type (ATCC 38606) strains of *P. nicotianae* were purchased from the ATCC (YA, USA). Two strains of *P. infestans*, PI0-1 (race 0) and PI1234-1 (race 1.2.3.4), were provided by Prof. K. Kawakita at the Graduate School of Bioagricultural Sciences, Nagoya University. The remaining 76 strains were obtained from the Biological Resource Centre, National Institute of Technology and Evaluation (NBRC, Chiba, Japan).

### Pairing of strains to determine the mating type

Each strain was paired with *P. nicotianae* ATCC 38607 (A1) or ATCC 38606 (A2) by the polycarbonate membrane method described by Ko^11^. Briefly, a loopful of *Phytophthora* strain was inoculated onto a Petri plate (ϕ 9 cm) containing 10% V8 juice (Campbell Soup Company, Camden, NJ), 0.02% CaCO_3_, and 2% agar, and incubated in an incubator (Eyela KCL-2000A, Tokyo Rikakikai Co., Ltd., Tokyo, Japan) at 25 °C and 60% humidity for 10 days, except for *P. infestans*, which was incubated at 20 °C. In the case of *Halophytophthora*, 10% V8 juice in 2% artificial sea water (TetraMarine Salt Pro) was used under the same conditions as those for the *Phytophthora* strains. A disc (ϕ 1.8 cm) of mycelia on agar was cut out of a colony and placed with the mycelial side up at the center of a Petri plate. A sterilized polycarbonate membrane filter (4.7 cm diameter, 0.2 μm, Advantec, Tokyo, Japan) was placed on the mycelia disk. A disk of the standard A1 or A2 strain of *P. nicotianae* was placed with the mycelial side down on top of the filter. Finally, the upper agar disk was covered with a micro cover glass (22 mm × 22 mm) to avoid desiccation. After incubation for 7 days, both agar disks were detached from the filter paper and the oospores formed in the entire area were observed under a microscope for evaluation according to four grades: ++, +, w (weak), and − (Table S1). *P. nicotianae* 38607 and 38606 were paired as controls in all experiments.

### Evaluation of hormone responsiveness

A loopful of mycelia of a *Phytophthora* strain was inoculated onto a Petri plate (ϕ 9 cm) containing 20% V8 juice, 0.3% CaCO_3_, and 2% agar, and incubated at 25 °C and 60% humidity for 10 days in the dark. A piece (3 mm × 3 mm) of the colony was then incubated on a Petri plate containing 10% V8 juice, 0.02% CaCO_3_, and 2% agar and incubated for 4 days at 25 °C and 60% humidity in the dark, except for *P. infestans*, which was incubated at 20 °C. For *Halophytophthora*, an artificial sea water-containing medium was used, as mentioned above. A solution of α1 in acetonitrile (100–300 ng 30 µL^−1^) was applied to a paper disc (0.7 mm thickness, ϕ 8 mm diameter, Advantec), which was dried *in vacuo* for 20 min and placed on the colony at a distance of 1.5 cm from the colony center. After incubating for an additional 3 days, the agar around the paper disc (ϕ 1.8 cm) was cut out and the total number of oospores formed in the entire area was counted under a microscope. Almost all oospores were formed within the area cut. The responsiveness to α1 was regarded as “ + ” when more than 10 oospores were observed.

The responsiveness to α2 was tested as follows. A piece (3 mm × 3 mm) of a colony of a *Phytophthora* strain was incubated for 8 days under the same conditions as those for the above experiment, except that the medium was adjusted to pH 8 by 6 M KOH. A solution of α2 in 95% ethanol (100–300 ng 5 µL^−1^) was applied to a mixed-cellulose ester membrane filter (No. A020A013A, ϕ 13 mm, 0.2 μm, White; Advantec), which was then dried at room temperature for 30 min and placed on the colony at a distance of 1.5 cm from the colony center. After incubation for 4 days, the agar around the membrane filter (ϕ 2.1 cm) was cut out and the total number of oospores formed in the entire area was counted under a microscope. The responsiveness was evaluated, as mentioned for α1 above.

### Quantitative analysis of α hormones by LC/MS

All *Phytophthora* and *Halophytophthora* strains were cultured in 20% V8 juice, except for the two strains of *Halophytophthora* (*H. vesicula* 33165 *and H. vesicula* 33166), which were cultured in 20% V8 juice containing 2% saline water. For the A2 and A1,A2 mating-type strains, the medium (1.6 L) was supplemented with phytol (2 mg L^−1^). For the A1 mating-type strains, a small piece of an A2 strain of the same species (or *P. nicotianae* ATCC 38606, if not available) was added to the medium (2.0 L). The homothallic strains were cultured in the medium (1.6 L) without the aforementioned additives. Unless otherwise noted, liquid culture was incubated for one week at 27 °C and 80 rpm, using a shaking incubator TB-98RS (Takasaki Scientific Instruments, Saitama, Japan). In the case of *P. infestans*, strains were cultured in 200 mL of the medium (in a 500 mL flask) or in 400 mL of the medium (in a 1 L flask) at 20 °C by using a shaking incubator (BR-33FL MR; Taitec, Saitama, Japan). Some strains that grew slowly were cultured for 11 days or two weeks. The mycelia were separated by suction filtration and the filtrate was extracted twice with 1 L ethyl acetate. The organic layers were combined, washed with water (200 mL), and concentrated *in vacuo*. The resulting brownish material was suspended in 70% (for A1 strains) or 80% (for A2 strains) aqueous acetonitrile (1 mL) and adsorbed onto an ODS cartridge [TOYOPAK ODS-M (0.3 g ODS); Tosoh, Tokyo, Japan], which was then eluted with 70% (or 80%) aqueous acetonitrile (0.5 mL, three times) to yield a 2.5 mL solution. A portion (1 or 0.2 μL) of the solution or standard hormone solutions (0.5 and 2 ng 2 μL^−1^ in 50% acetonitrile) was injected into an Agilent 1100 High-Performance Liquid Chromatography (HPLC) system (Hewlett Packard, CA) connected to a Mariner Biospectrometry Workstation (Applied Biosystems, CA). The HPLC conditions were as follows: column, Unison UK-C8 (ϕ 2.0 × 75 mm, Imtakt, Kyoto, Japan); mobile phase: 38% acetonitrile–0.1% HCOOH for α1 or 48% acetonitrile–0.3 mM HCOONa–0.3 mM HCOOH for α2; flow rate, 0.1 mL min^−1^. The MS conditions were as follows: ionization and detection, ESI-TOF; positive mode; flow rate, 5 μL min^−1^ (split ratio of LC/MS, 20:1). The hormones α1 and α2 were detected on an extracted ion chromatograph (XIC) by using *m/z* 309.3 [M-2H_2_O + H]^+^ and *m/z* 351.3 [M + Na]^+^ ions at approximately 7.8 and 7.6 min, respectively. The peak areas were used for quantitative analysis. The detection limit was 0.3 and 0.16 μg L^−1^ broth^−1^ for α1 and α2, respectively, based on the standard curves (Fig. S1).

### Quantitative analysis of time-dependent production of the mating hormone in 20% V8 juice medium

After preincubation of *P. nicotianae* ATCC 38607, *P. cinnamomi* NBRC 33180, *P. nicotianae* NBRC 33190, and *P. cryptogea* NBRC 32326 for 7 days and *P. capsici* NBRC 8386 for 10 days by the method mentioned in Section 4.3, two pieces (10 mm × 10 mm) of a colony were cut out and inoculated into a 500 mL Erlenmeyer flask containing 200 mL of 20% V8 liquid medium supplemented with phytol (1 mg L^−1^ for A2 mating type) or α2 (50 μg L^−1^ for A1 mating type). The flask was incubated at 27 °C and 80 rpm for 7 days in the dark by a shaking incubator (TB-98RS). During cultivation, a 5 mL portion of the culture broth was tapped from the flask daily, centrifuged at 4,000 rpm for 5 min, and the supernatant was directly adsorbed onto an ODS cartridge [TOYOPAK ODS-S (90 mg ODS); Tosoh, Tokyo, Japan], which was washed with water (1.5 mL) and then eluted with 2.5 mL 80% aqueous acetonitrile. The eluate with 80% acetonitrile was diluted to a total volume of 5 mL with 20% aqueous acetonitrile, following which a 5 μL portion of the sample solution or a standard solution (10 pg μL^−1^ of α1 or α2 in 50% aqueous acetonitrile) was injected into an Agilent 1200 HPLC system (Hewlett Packard) equipped with an HCTplus mass spectrometer (Bruker Daltonics, Billerica, MA). HPLC was performed under the following conditions: column, Cadenza CD-C18 (ϕ 2.0 × 75 mm, Imtakt); mobile phase: 34% acetonitrile for α1 and 45% acetonitrile for α2; flow rate, 0.2 mL min^−1^. The MS conditions were as follows: ionization and detection, electrospray ionization-ionTrap (ESI-IT); positive mode. The hormones α1 and α2 were detected on an extracted ion chromatograph (EIC) by using *m/z* 367.2 [M + Na]^+^ and *m/z* 351.2 [M + Na]^+^ ions at approximately 6.7 and 6.4 min, respectively. The production of mating hormones was calculated from the peak areas of each standard. The detection limit was 0.2 and 1 μg L^−1^ broth^−1^ for α1 and α2, respectively, based on the standard curves (Fig. S2).

### Mating hormone production in Czapek-Dox liquid media

Two pieces (10 mm × 10 mm) of a *Phytophthora* colony were inoculated into 200 mL of Czapek-Dox liquid medium (3 g L^−1^ of NaNO_3_, 1 g L^−1^ of K_2_HPO_4_, 0.5 g L^−1^ of MgSO_4_ 7H_2_O, 0.5 g L^−1^ of KCl, 0.01 g L^−1^ of FeSO_4_ 7H_2_O, and 30 g L^−1^ of sucrose), which was supplemented with phytol (1 mg L^−1^). Flasks were incubated at 27 °C and 80 rpm for 7 days in the dark by a shaking incubator TB-98RS. The production of mating hormones was quantified by the same method as mentioned above. The mycelia were obtained by suction filtration and washed twice with Milli-Q water. After lyophilization, the weight of dried mycelia was measured.

### Preparation of polar and non-polar fractions from V8 juice

A volume of 2 L of V8 juice was separated into the supernatant and precipitated by centrifugation at 7,000 g for 5 min. The precipitate was washed with the same volume of water and the second supernatant was obtained by centrifugation. The supernatants were combined and chromatographed on an ODS column [Cosmosil 140C18-OPN, 10 (i.d.) × 70 cm, Nacalai Tesque, Kyoto, Japan] using Milli-Q water, MeOH, and 50% MeOH in CHCl_3_. The fraction eluted with Milli-Q water was diluted with Milli-Q water to 10 L (20% V8 juice equivalent) and used as the polar (P) medium. The fractions eluted with organic solvents (ODS-adsorbed fractions) were combined. The abovementioned precipitate (juice dregs) was suspended in EtOAc and stirred at room temperature for 2 h (1 L, three times). After the filtration, EtOAc solutions were concentrated to provide a residue. This extract was combined with the ODS-adsorbed fractions and concentrated to obtain 3.3 g of the non-polar fraction, which was dissolved in EtOH, and water was added to prepare the non-polar (NP) medium.

## Electronic supplementary material

Supplementary Information
