# Supplementary material for: Universality of the Phytophthora mating hormones and diversity of their production profile
Source: Sci Rep. 2017 Jul 10;7:5007. doi: 10.1038/s41598-017-05380-3 (PMC5504046; doi:10.1038/s41598-017-05380-3)
Supplement: Supplementary file 1 — Supplementary Information [file 41598_2017_5380_MOESM1_ESM.pdf]

Supplementary Information

Universality of the *Phytophthora* mating hormones and diversity of their production profile

Tomohiko Tomura, Shylaja D. Molli, Ryo Murata, and Makoto Ojika*
Graduate School of Bioagricultural Sciences, Nagoya University, Nagoya 464-8601, Japan

Contents

Figure S1. LC/MS analysis and standard curves of the mating hormones with a Mariner Biospectrometry Workstation

2

Figure S2. LC/MS analysis and standard curves of the mating hormones with an HCTplus mass spectrometer

3

Table S1. Mating type determination by polycarbonate membrane co-culture between the standard and tested strains of *Phytophthora*

4

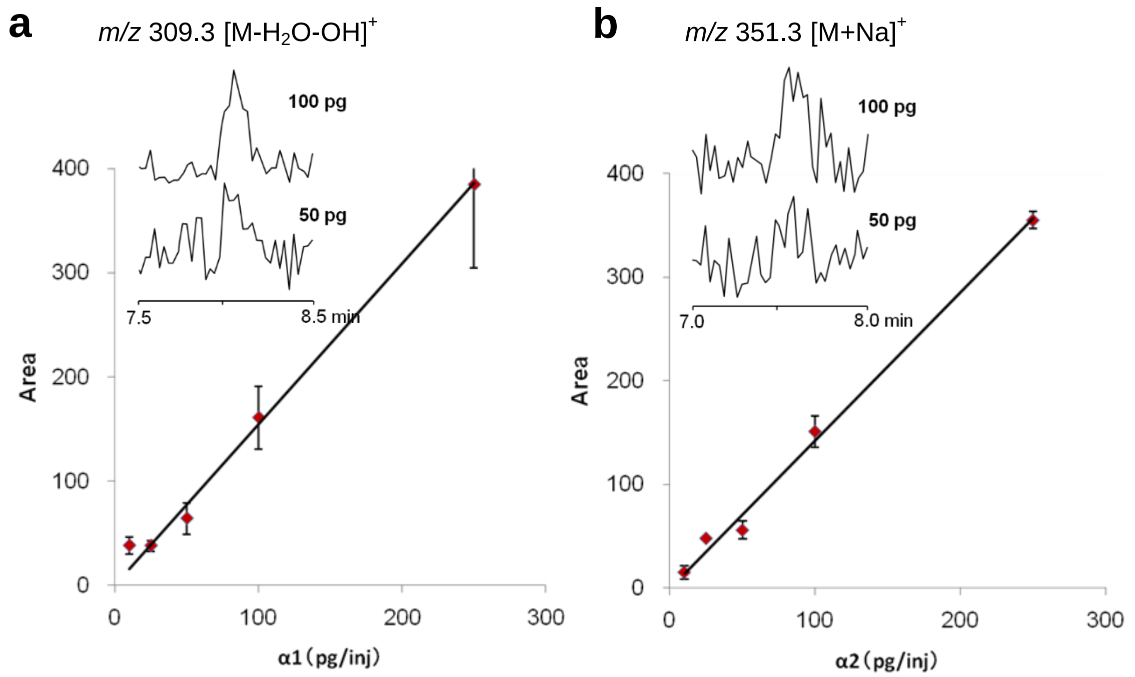

Figure S1. LC/MS analysis and standard curves of the mating hormones with a Mariner Biospectrometry Workstation.

Standard curves for the quantitative analysis of α1 (**a**) and α2 (**b**) were obtained by using peak areas of extracted ion chromatographs, which were obtained by using the particular pseudo-molecular ions indicated in the top. Two chromatographs obtained by injecting 50 and 100 pg are exemplified in the upper left for each standard curve. Data are the mean ± SE of three experiments.

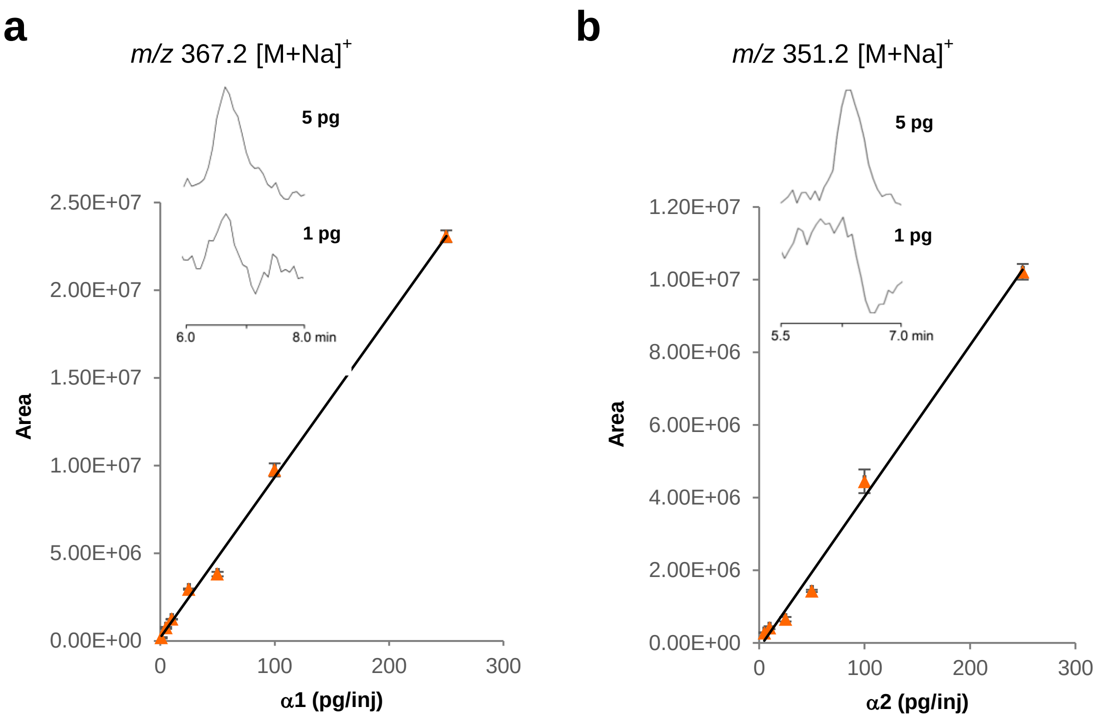

Figure S2. LC/MS analysis and standard curves of the mating hormones with an HCTplus mass spectrometer.

Standard curves for the quantitative analysis of α1 (**a**) and α2 (**b**) were obtained by using peak areas of extracted ion chromatographs, which were obtained by using the particular pseudo-molecular ions indicated in the top. Two chromatographs obtained by injecting 1 and 5 pg of hormones are exemplified in the upper left for each standard curve. Data are the mean ± SE of three experiments.

Species	Strain No.[a]	mating type	Oospores induced by[b]		Oospore induction on[b]	
			A1 (α1 reception)	A2 (α2 reception)	A1 (α2 production)	A2 (α1 production)
Phytophthora cactorum	30474	homo	++	++	++	-
Phytophthora cactorum	32191	homo	++	++	++	-
Phytophthora cactorum	32192	homo	++	++	w	-
Phytophthora cactorum	32193	homo	++	++	++	w
Phytophthora cactorum	32194	homo	++	++	w	-
Phytophthora cambivora	30471	A1	-	-	-	+
Phytophthora cambivora	30472	A2	w	-	++	w
Phytophthora capsici	8386	A2	-	-	++	w
Phytophthora capsici	30696	A1	-	++	-	++
Phytophthora capsici	30697	A2	++	-	++	-
Phytophthora capsici	30698	A2	-	-	++	-
Phytophthora capsici	30699	A1	-	-	w	++
Phytophthora capsici	31400	A1,A2	-	-	w	w
Phytophthora capsici	31402	A1	-	+	-	++
Phytophthora cinnamomi	33180	A1	-	+	-	+
Phytophthora cinnamomi	33181	A1,A2	+	-	++	+
Phytophthora cinnamomi	33182	A1	-	+	w	+
Phytophthora cinnamomi	33183	A1,A2	w	-	++	+
Phytophthora citrophthora	31408	A0	-	-	-	-
Phytophthora citrophthora	31410	A1,A2	-	-	w	w
Phytophthora colocasiae	30695	A2	-	-	++	-
Phytophthora cryptogea	31411	A1	-	-	-	+
Phytophthora cryptogea	31412	A1	-	-	-	+
Phytophthora cryptogea	31622	A2	-	-	++	-
Phytophthora cryptogea	32325	A1,A2	-	-	++	+
Phytophthora cryptogea	32326	A2	-	-	++	w
Phytophthora humicola	32771	homo	++	++	-	++
Phytophthora infestans	9173	A1	-	-	w	+
Phytophthora infestans	9174	A1,A2	-	-	+	+
Phytophthora infestans	PI 0-1	A1	-	-	-	++
Phytophthora infestans	PI 1234-1	A2	-	-	++	-
Phytophthora katsurae	9753	A2	-	-	w	-
Phytophthora katsurae	30433	A2	-	-	w	-
Phytophthora katsurae	30434	homo	+	+	w	-
Phytophthora katsurae	30435	homo	++	+	+	-
Phytophthora megasperma	31624	homo	++	++	+	-
Phytophthora megasperma	32174	homo	++	++	w	-
Phytophthora megasperma	32175	homo	++	++	w	-
Phytophthora megasperma	32176	homo	++	++	w	-
Phytophthora melonis	31413	A1	-	+	-	+
Phytophthora melonis	31414	A1,A2	-	-	++	+
Phytophthora melonis	31415	A2	-	-	++	-
Phytophthora nicotianae	9049	A2	w	-	++	-
Phytophthora nicotianae	33190	A2	++	-	++	-
Phytophthora nicotianae	33191	A1	-	++	-	++
Phytophthora nicotianae	33192	A2	-	-	++	-
Phytophthora nicotianae	33193	A2	++	-	++	-
Phytophthora nicotianae	ATCC 38606	A2	++	-	++	-
Phytophthora nicotianae	ATCC 38607	A1	-	++	-	++
Phytophthora nicotianae var. *nicotianae*[c]	4873	A2	-	-	+	-
Phytophthora nicotianae var. *parasitica*[c]	30595	A2	++	-	++	-

Phytophthora nicotianae var. *parasitica*[c]	31416	A2	++	-	++	-
Phytophthora nicotianae var. *parasitica*[c]	31419	A2	+	-	++	-
Phytophthora nicotianae var. *parasitica*[c]	31423	A1	-	++	-	++
Phytophthora nicotianae var. *parasitica*[c]	31425	A1	-	++	-	++
Phytophthora palmivora	9755	A2	-	-	++	-
Phytophthora palmivora	30285	A1	-	-	-	++
Phytophthora palmivora	31428	A1	-	-	-	++
Phytophthora porri	30417	A0	-	-	-	-
Phytophthora porri	32963	A0	-	-	-	-
Phytophthora sojae	31014	homo	++	++	-	+
Phytophthora sojae	31015	homo	++	++	-	++
Phytophthora sojae	101543	homo	++	++	-	++
Phytophthora sojae	101544	homo	++	++	-	+
Phytophthora sojae	101545	homo	++	++	-	+
Phytophthora sojae	101546	homo	++	++	-	++
Phytophthora sojae	101547	homo	++	++	-	++
Phytophthora sojae	105919	homo	++	++	-	+
Phytophthora vignae	30473	homo	++	++	w	++
Phytophthora vignae	30613	homo	++	++	w	++
Phytophthora sp.	32716	A1	-	-	-	w
Halophytophthora operculata	32865	A2	-	-	+	-
Halophytophthora vesicula	32216	A2	-	-	w	-
Halophytophthora vesicula	32444	A2	-	-	w	-
Halophytophthora vesicula	32445	A2	-	-	w	-
Halophytophthora vesicula	33128	A2	-	-	w	-
Halophytophthora vesicula	33164	A2	-	-	w	-
Halophytophthora vesicula	33165	A2	-	-	++	-
Halophytophthora vesicula	33166	A2	-	-	+	-
Halophytophthora vesicula	33265	A2	-	-	w	-

Table S1. Mating type determination by polycarbonate membrane co-culture between the standard and tested strains of *Phytophthora*.

[a]The strain numbers in only numerals are NBRC numbers (30474 means NBRC 30474). [b]Relative oospore formation activity: "++" high; "+" regular; "w" weak; "-" almost no. In the oospore induction on the tester strains (A1/A2), "w" of the combination of w/++ (or w/+) is regarded as negative (-) activity, resulting in the judgement as A1 or A2 type, whereas the strains with the combination of w/w were judged as A1,A1 type. [c]These variants are regarded as synonyms of *P. nicotianae* in the NBRC catalog (http://www.nbrc.nite.go.jp).
